# KUS121 attenuates the progression of monosodium iodoacetate-induced osteoarthritis in rats

**DOI:** 10.1038/s41598-021-95173-6

**Published:** 2021-08-02

**Authors:** Sachiko Iwai, Hanako O. Ikeda, Hisashi Mera, Kohei Nishitani, Motoo Saito, Akitaka Tsujikawa, Akira Kakizuka

**Affiliations:** 1 Department of Ophthalmology and Visual Sciences, Graduate School of Medicine, Kyoto University, Kyoto, Japan; 2 Uonuma Institute of Community Medicine, Niigata University Medical and Dental Hospital, Niigata, Japan; 3 Department of Orthopedic Surgery, Graduate School of Medicine, Kyoto University, Kyoto, Japan; 4 Laboratory of Functional Biology, Graduate School of Biostudies, Kyoto University, Kyoto, Japan

**Keywords:** Osteoarthritis, Molecularly targeted therapy

## Abstract

Currently there is no effective treatment available for osteoarthritis (OA). We have recently developed Kyoto University Substances (KUSs), ATPase inhibitors specific for valosin-containing protein (VCP), as a novel class of medicine for cellular protection. KUSs suppressed intracellular ATP depletion, endoplasmic reticulum (ER) stress, and cell death. In this study, we investigated the effects of KUS121 on chondrocyte cell death. In cultured chondrocytes differentiated from ATDC5 cells, KUS121 suppressed the decline in ATP levels and apoptotic cell death under stress conditions induced by TNFα. KUS121 ameliorated TNFα-induced reduction of gene expression in chondrocytes, such as *Sox9* and *Col2α*. KUS121 also suppressed ER stress and cell death in chondrocytes under tunicamycin load. Furthermore, intraperitoneal administration of KUS121 in vivo suppressed chondrocyte loss and proteoglycan reduction in knee joints of a monosodium iodoacetate-induced OA rat model. Moreover, intra-articular administration of KUS121 more prominently reduced the apoptosis of the affected chondrocytes. These results demonstrate that KUS121 protects chondrocytes from stress-induced cell death in vitro and in vivo, and indicate that KUS121 is a promising novel therapeutic agent to prevent the progression of OA.

## Introduction

Osteoarthritis (OA) is a common degenerative joint disease observed in the elderly, and the pain is the greatest burden to the patients. Approximately 10 to 15% of individuals worldwide is estimated to suffer from OA, making it a great burden to societies. Various factors, such as aging, obesity, and excess exercise, contribute to the development of OA^1,2^, and more directly, chondrocyte death is proposedly to be the most important cause^3^. Chondrocyte death has characteristics of apoptosis, and inflammatory cytokines, e.g. tumor necrosis factor-alpha (TNF-α), have been detected in the affected joints of patients with OA^4,5^. TNF-α exposure reduces cell viability and increases apoptosis in many types of cells, including ATDC5 cells, a mouse chondrogenic cell line derived from teratocarcinoma^6–9^. In addition, increased ER stress is usually observed in joint tissues isolated from patients with OA^10,11^.

Valosin-containing protein (VCP), a member of AAA ATPases, is the most abundantly expressed soluble ATPase and is essential for many cellular processes, e.g. ER-associated degradation, DNA damage response, and cell cycle^12^. We observed that the loss-of-function of ter94 (*Drosophila* VCP) mitigates neurodegeneration in a *Drosophila* model of polyglutamine diseases, and thus developed new chemical compounds that inhibit the ATPase activity but not the other cellular functions of VCP. We named these compounds “Kyoto University Substances (KUSs)” or “VCP modulators”^13^. KUS121 exerts potent protective effects on retinal neuronal cells, including retinal ganglion cells and photoreceptors, dopaminergic neurons in the midbrain, cardio-muscles, and cerebral neurons, in animal models of different diseases^14–17^. These protective effects are profoundly coupled with their ability to ameliorate decreased ATP levels and ER stress in the cells. Given that chondrocyte cell death in OA is attributable to ER stress, KUS121 could be a new therapeutic agent for OA. We recently reported the therapeutic effects of KUS121 on post-traumatic cartilage damage in rats^18^. In this study, we aimed to examine the link between ER stress and chondrocyte cell death, as well as the effects of KUS121 on these phenomena in vitro and in vivo in monosodium iodoacetate (MIA)-induced rat OA models^19,20^.

## Results

### KUS121 suppresses TNF-α mediated apoptosis in chondrocytes differentiated from ATDC5 cells

**Figure 1:**
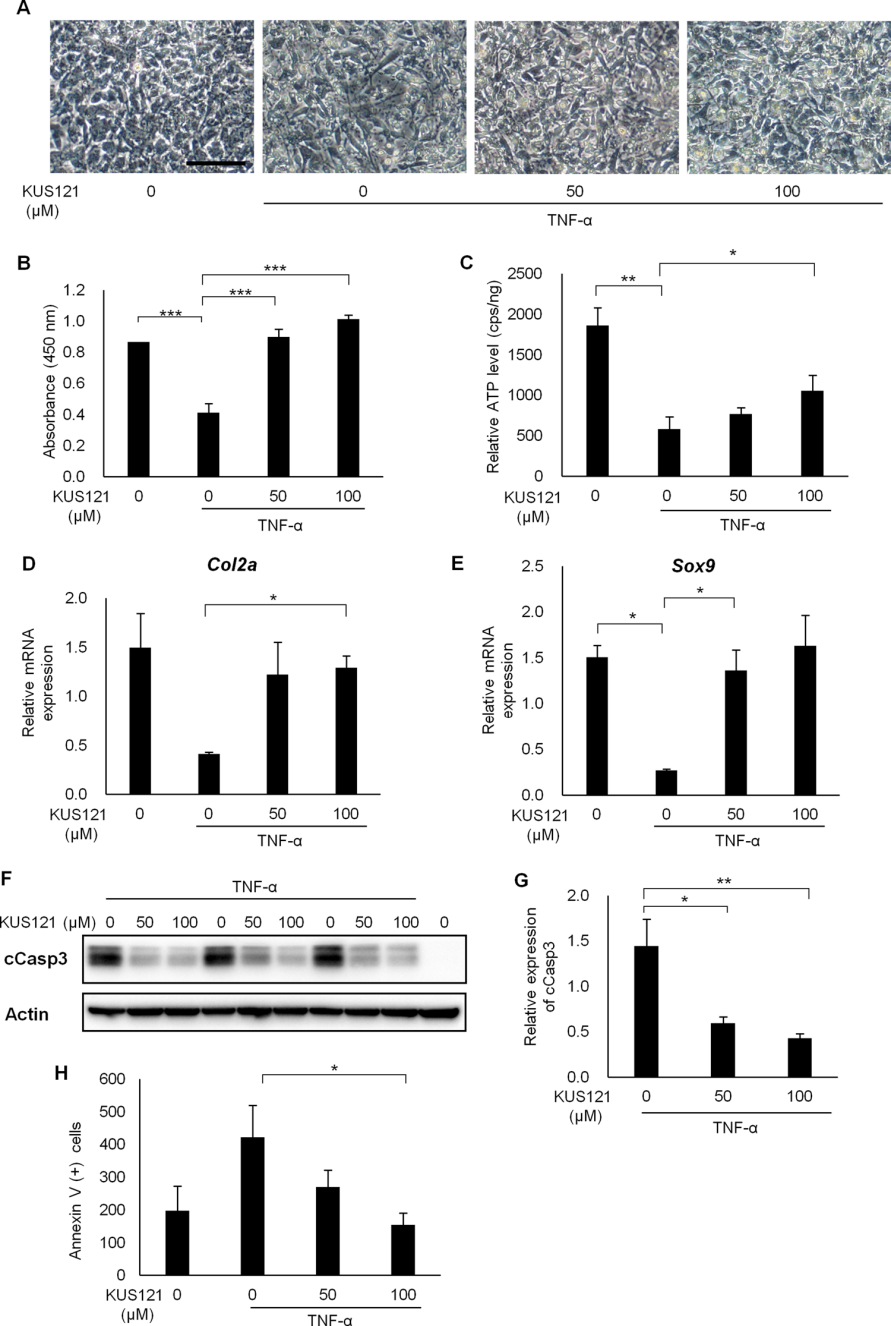
KUS121 suppresses TNF-α-induced cell death in differentiated ATDC5 cells. (**A**–**C**) Differentiated ATDC5 cells, cultured with tumor necrosis factor-alpha (TNF-α) (10 ng/mL) and KUS121 (0, 50 or 100 µM) for 48 h. (**A**) Brightfield images of the cells. Scale bar, 50 µm. (**B**) Water-soluble tetrazolium salt (WST) values, which reflect the relative live cell number, are shown as optical density at 450 nm. ***p < 0.001, vs TNF-α without KUS121, Dunnett T3 test, N = 6. (**C**) Intracellular ATP levels of the differentiated ATDC5 cells were measured with luciferase assays. The total protein content of cell lysate in each well was used for standardization. *p < 0.05, vs TNF-α without KUS121, Dunnett T3 test, N = 5. (**D**,**E**) Real-time PCR analysis of differentiated ATDC5 cells, treated with TNF-α (20 ng/mL) and KUS121 (0, 50 or 100 µM) for 24 h. mRNA expression levels of *Col2a* and *Sox9* were normalized to *Gapdh* and relative expression levels are shown. *p < 0.05, vs TNF-α without KUS121, Dunnett T3, N = 3. (**F**,**G**) Western blot analysis of differentiated ATDC5 cells, treated with TNF-α (10 ng/mL) and KUS121 (0, 50 or 100 µM) for 48 h. Actin was used as a loading control. (**G**) The expression of cleaved caspase-3 (cCasp-3) was quantified and relative values were normalized to that of actin. Full-length blots are presented in Supplementary Fig. 2A. *p < 0.05 and **p < 0.01, vs TNF-α without KUS121, Dunnett T3 test, N = 3. (**H**) Differentiated ATDC5 cells cultured with TNF-α (20 ng/mL) and KUS121 (0, 50 or 100 µM) for 48 h. Apoptotic cells were stained with Annexin V and analyzed via fluorescence-activated cell sorting. The Annexin V-positive cell number was determined (see Supplementary Fig. 3A). *p < 0.05 vs TNF-α without KUS121, Dunnett T3 test, N = 4. Error bars indicate standard deviation.

We first examined the effects of KUS121 on cell death in cultured chondrocytes. ATDC5 mouse chondrogenic cells were differentiated into cartilage cells and the differentiation was confirmed by Alcian blue staining (Supplementary Fig. 1). Differentiated cartilage cells were treated with TNF-α. We observed that treatment with 10 ng/mL TNF-α changed the cell shape and density, as well as viability, compared to non-treated controls 48 h after treatment. In contrast, co-treatment with 10 ng/mL TNF-α and 50 or 100 μM KUS121 recovered the cell shape and density (Fig. 1A). The cell viability was also recovered upon KUS121 co-treatment in a dose-dependent manner (Fig. 1B). Intracellular ATP levels decreased in TNF-α-treated cells and were marginally and significantly recovered upon treatment with 100 μM KUS121 (P < 0.05, respectively, Fig. 1C). *Col2a* and *Sox9* mRNA expression*,* expressed in differentiated chondrocytes^21,22^, was reduced in TNF-α treated cells, and restored by the addition of KUS121 (Fig. 1D,E). These results demonstrated that KUS121 protected chondrocytes from TNF-α-induced cell damage.

To evaluate the characteristics of TNF-α-mediated cell death, cleaved caspase-3 (cCasp3) expression, an apoptotic indicator, was measured using western blotting. cCasp3 expression was upregulated upon TNF-α treatment and reduced upon KUS121 treatment (Fig. 1F,G). Phosphatidylserine (PS) is exposed to the outside of the cell membrane during apoptosis. Apoptosis was detected using Annexin V, which binds to PS. Fluorescence-activated cell sorting (FACS) analysis showed that the number of annexin V-stained cells were reduced by KUS121 treatment (Fig. 1H, Supplementary Fig. 3A). The ratio of single stranded DNA (ssDNA)-positive cells was also lower in KUS121-treated cells (Supplementary Fig. 3B,C). These results indicated that KUS121 suppressed apoptotic cell death in TNF-α-treated chondrocytes.

### KUS121 protects chondrocytes under ER stress

**Figure 2:**
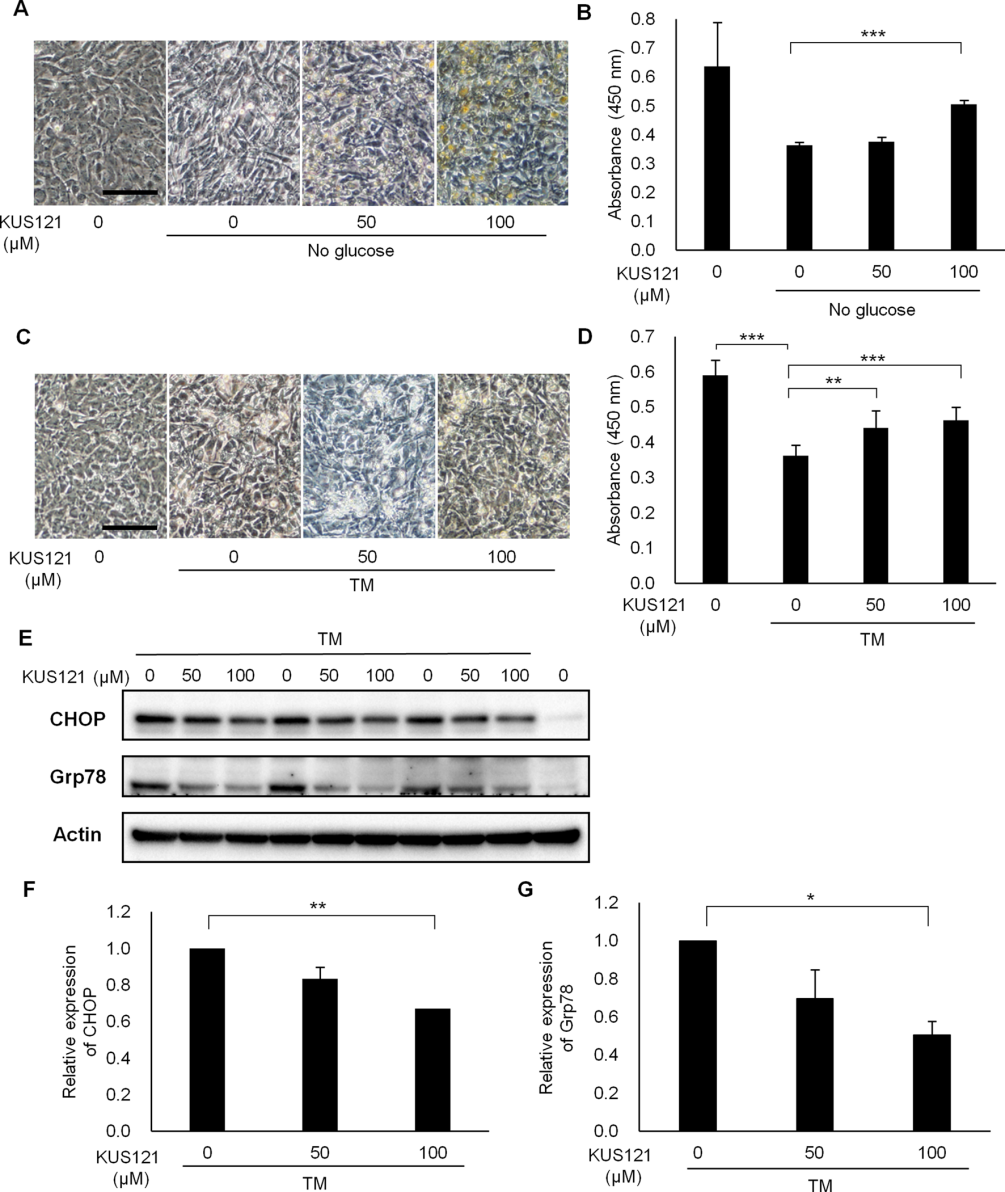
KUS121 reduces ER stress in ATDC5 cells. (**A**,**B**) Differentiated ATDC5 cells, cultured with KUS121 (0, 50 or 100 µM) for 5 days under glucose-free conditions. (**A**) Brightfield images of the cells. (**B**) Water-soluble tetrazolium (WST) assay results of ATDC5 cells are shown as optical density values at 450 nm. ***p < 0.001, vs no glucose without KUS121, Dunnett T3 test, N = 5. (**C**,**D**) Differentiated ATDC5 cells were cultured with tunicamycin (TM, 0.2 µg/mL) and KUS121 (0, 50 or 100 µM) for 40 h. (**C**) Brightfield images of the cells. (**D**) WST assay results of ATDC5 cells are shown as optical density values at 450 nm. **p < 0.01 and ***p < 0.001, vs TM without KUS121, Dunnett T3 test, N = 9. (**E**–**G**) Western blot analysis of differentiated ATDC5 cells, treated with TM (3 µg/mL) and KUS121 (0, 50 or 100 µM) for 6 h. (**F**,**G**) Expression levels of C/EBP-homologous protein (CHOP) and 78 kDa glucose-regulated protein (Grp78) were quantified and relative values were normalized to those of actin. Full-length blots are presented in Supplementary Fig. 2B. *p < 0.05 and **p < 0.01, vs TM without KUS121, Dunnett T3 test, N = 3. Error bars indicate standard deviation. Scale bars, 50 µm in (**A**) and (**C**).

We next examined the protective effects of KUS121 on the chondrocytes, which were differentiated from ATDC5 cells, under ER stress. Chondrocytes were cultured for 5 days without glucose or 40 h with tunicamycin (TM)^13^. Expressions of ER stress markers C/EBP-homologous protein (CHOP) and Grp78 were upregulated under these conditions. The addition of KUS121 suppressed the expressions of CHOP and Grp78 in TM-treated chondrocytes (Fig. 2E–G).

We confirmed that these stresses caused changes in cell morphology and cell death in chondrocytes. The addition of KUS121 prevented the changes in cell morphology (Fig. 2A,C). Likewise, the addition of KUS121 prevented cell death under these conditions in a dose-dependent manner (Fig. 2B,D).

These results indicated that KUS121 suppressed ER stress in chondrocytes and promoted their survival under ER stress-inducing conditions.

### KUS121 treatment restores histopathological features of knee joint tissue in an OA rat model

**Figure 3:**
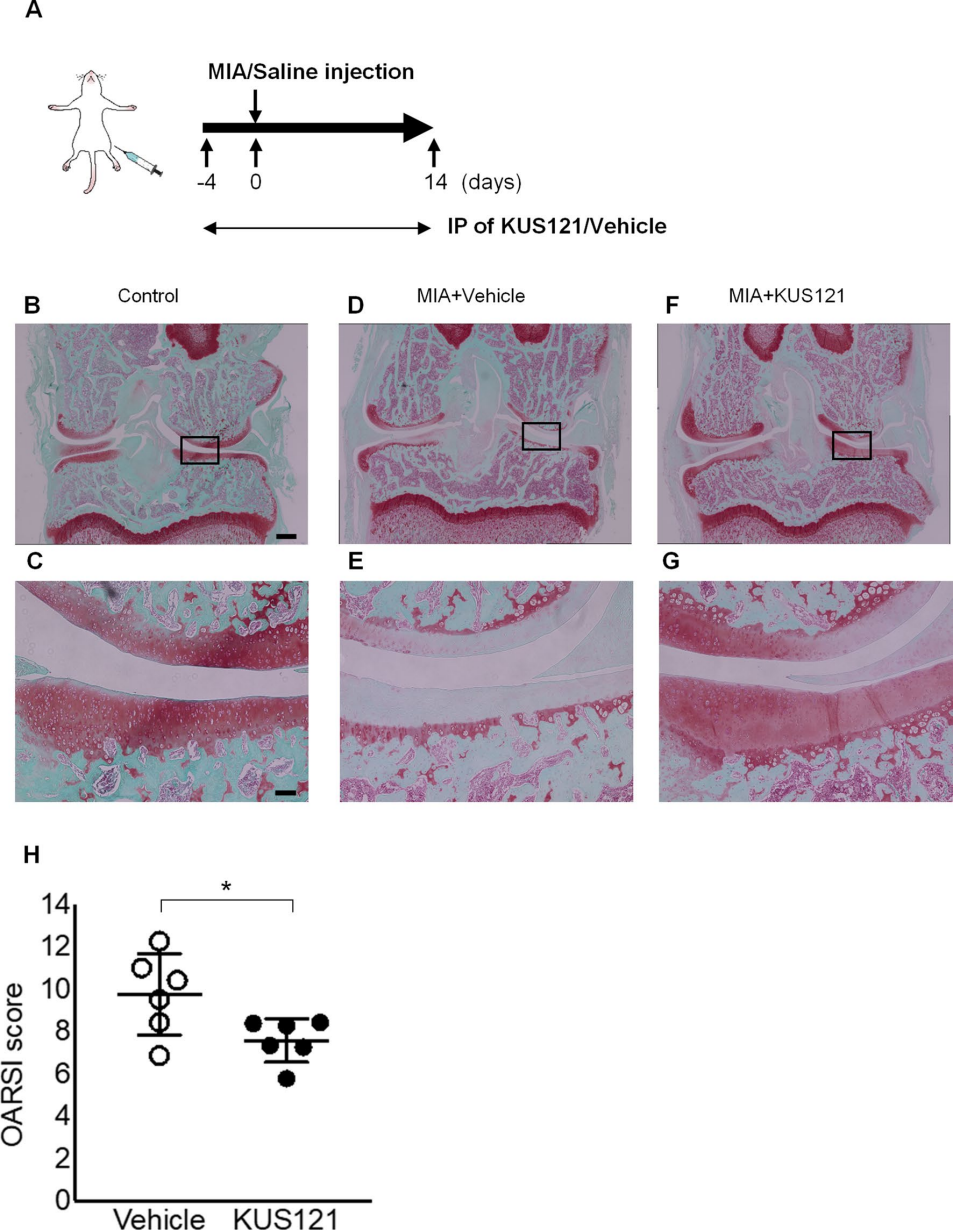
Efficacy of KUS121 in monosodium iodoacetate-induced osteoarthritis rat model. (**A**) Monosodium iodoacetate (MIA, 0.5 mg) was intra-articularly injected into the knee joint of rats to create an osteoarthritis (OA) model. KUS121 was administered daily intraperitoneally (IP) from 4 days before until 14 days after MIA injection. (**B**–**G**) Knee joints of OA rats treated with either KUS121 (**F**,**G**) or vehicle (**D**,**E**) were stained with Safranin-O and Fast green. (**C**,**E**,**G**) Show magnified views in (**B**,**D**,**F**). Scale bars, 600 µm in (**B**,**D**,**F**); 100 µm in C, E, G. (**H**) The stained knee joints were graded on a scale of 0–24 using the Osteoarthritis Research Society International (OARSI) scoring system. Independent data points for OARSI scores with mean ± standard deviation are shown. *p < 0.05, vs MIA without KUS121, Mann–Whitney U test, N = 6.

Next, we investigated the protective effects of KUS121 on cartilage tissue in vivo in a rat knee OA model, which was established via the injection of monosodium iodoacetate (MIA) into articular knee joints. KUS121 was intraperitoneally administered once a day from 4 days before to 14 days after MIA injection (Fig. 3A). The knee joints were extracted and stained with Safranin-O/fast green to evaluate the cartilage of the femur and tibia (Fig. 3B–G). The control group (intra-articular saline injection) showed normal chondrocyte morphology with dense proteoglycan staining (Fig. 3B,C), and the MIA-injected group manifested marked loss of chondrocytes and reduced proteoglycan staining, showing OA-like irregular morphology (Fig. 3D,E). It was notable that in the KUS121-treated group, many chondrocytes remained and the irregular morphology was improved, indicating that the cartilage damage in the OA model was visibly restored (Fig. 3F,G). The degree of degeneration of knee joints was quantified using the Osteoarthritis Research Society International (OARSI) score. The OARSI score consisted of six grades (severities) and four stages (extents), and the total score (0–24 points) was calculated by combining each score; the higher the score, the more advanced the cartilage degeneration. The average OARSI score of the KUS121-treated group was 7.6 ± 1.0 and it was significantly lower than that in the MIA-treated group (9.7 ± 1.9) (Fig. 3H). These data indicated that systemic KUS121 administration prevented cartilage damage in vivo.

### Intra-articular administration of KUS121 attenuates apoptosis of chondrocytes in OA rats

**Figure 4:**
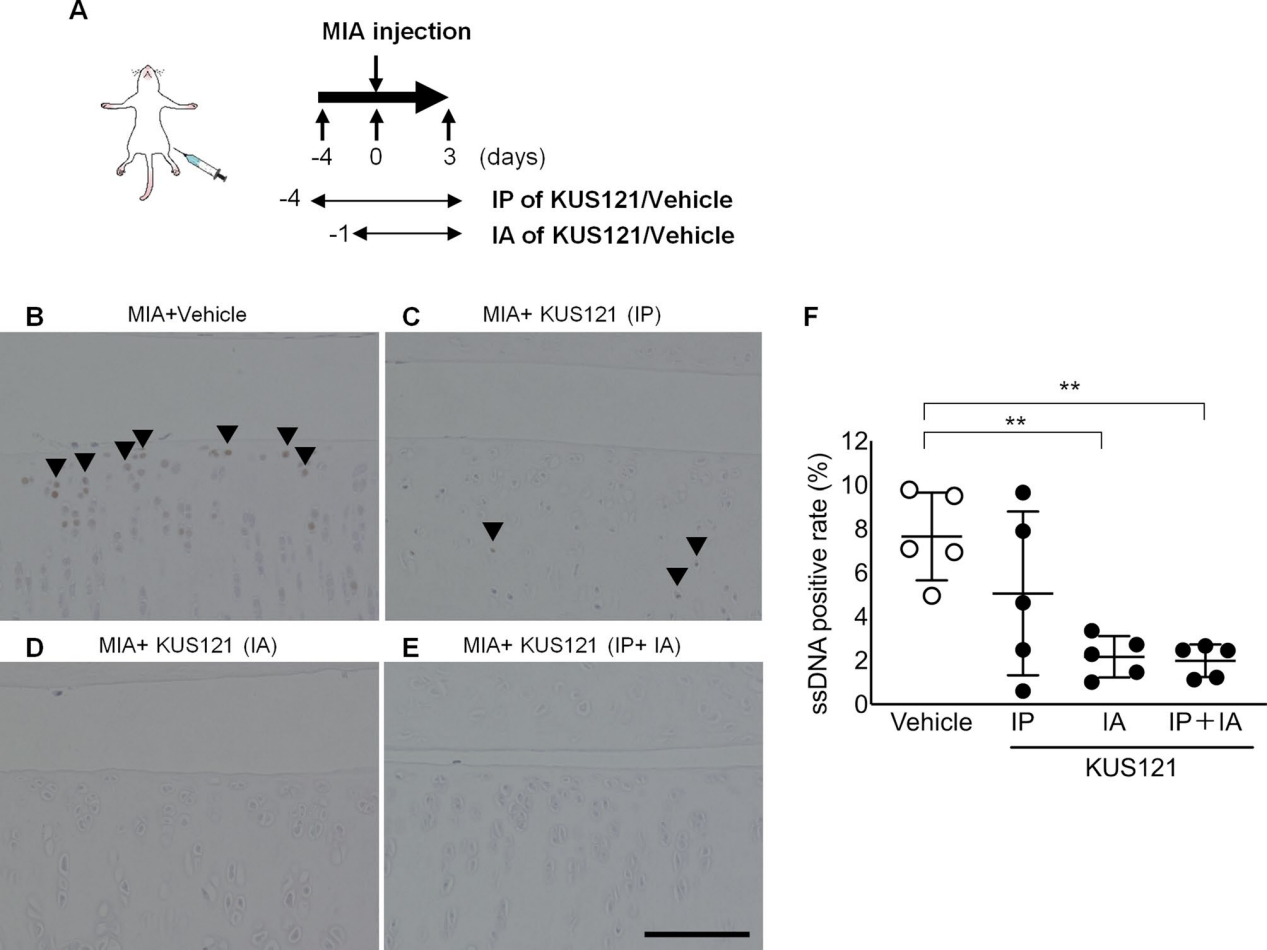
Administration of KUS121 reduces chondrocyte apoptosis in rats. (**A**) Monosodium iodoacetate (MIA, 0.5 mg) was intra-articularly injected into the knee joint of rats. KUS121 was administered intraperitoneally (IP, 50 mg/kg/day), intra-articularly (IA, 500 µg/day), or both. (**B**–**E**) Knee joints treated with either KUS121 or vehicle were stained with an anti-ssDNA antibody. Scale bar, 100 µm. Black triangles indicate ssDNA-positive apoptotic chondrocytes. (**F**) The ssDNA-positivity was calculated as the number of ssDNA-positive cells divided by the total number of chondrocytes above the Tidemark. Independent data points for ssDNA-positivity with mean ± standard deviation are shown. **p < 0.01, vs MIA without KUS121, Dunnett T3 test, N = 5.

We next evaluated the efficacy of KUS121 on MIA-induced knee osteoarthritis via direct intra-articular administration of KUS121, as this is the traditional clinical protocol. The rats were divided into four groups as follows: MIA + vehicle group; MIA + intraperitoneal administration of KUS121 group (IP); MIA + intra-articular administration of KUS121 group (IA); MIA + intraperitoneal and intra-articular administration of KUS121 group (IP + IA) (Fig. 4A). We evaluated apoptotic cells in the knee joints 3 days after MIA injection via ssDNA staining. (Fig. 4B–E). ssDNA-positive cell number only marginally decreased in the IP group, compared to the vehicle group. However, we observed a significant decrease in the number of apoptotic cells in the IA group, which was not further enhanced in the IP + IA group (Fig. 4F).

These results indicated that KUS121 had increased efficacy against OA progression and intra-articular administration might be the best way of KUS121 administration for OA treatment.

## Discussion

In this study, we elucidated that KUS121 can suppress cell death induced by TNF-α in chondrocytes differentiated from ATDC5 cells. More specifically, we showed that KUS121 protects ATDC5-derived chondrocytes from TNF-α-induced cell death and ATP depletion. Expressions of *Sox9* and *Col2a* were maintained by KUS121 at normal levels even in the presence of TNF-α. Furthermore, we revealed that the expression of cCasp3, an apoptosis marker, was induced by TNF-α in chondrocytes and was significantly suppressed by the addition of KUS121. In ATDC5-derived chondrocytes under ER stress-inducing conditions, KUS121 suppressed the ER stress, suppressing the upregulation of CHOP and Grp78 and cell death. Intraperitoneal and more prominent intra-articular administration of KUS121 attenuated disruption of the knee joint and suppressed apoptosis of affected chondrocytes in vivo in an MIA-induced OA rat model.

Due to the increase in elderly and obese populations, OA is becoming more prevalent and is currently estimated to affect 250 million people worldwide^1^. Non-surgical treatment for OA is rarely available and no fundamental treatment exists to prevent the progression^2,3^. TNF-α overexpression is commonly observed in the joints of patients with OA and is believed to play essential roles in its progression^4,5^.

We have developed KUSs as specific inhibitors of VCP ATPase activity^13^. KUSs suppress cell death under various stresses through the suppression of intracellular decrease in ATP levels and ER stress. Systemic or local administration of KUSs suppresses disease progression or deterioration in animal models of ocular diseases^14–17^, ischemic heart disease^23^, ischemic stroke^24^, and Parkinson’s disease^25^. We recently reported the therapeutic effects of KUS121 on post-traumatic cartilage damage in rats^18^. Among KUSs, KUS121, which has strong protective effects, was safe and effective in a phase 1/2 clinical trial in patients with central retinal artery occlusion^26^.

In this study, we focused on arthritis exacerbated by inflammation and stress conditions that may be caused by mechanical damage, such as trauma. KUS121 suppressed the apoptosis of ATDC5-derived chondrocytes associated with TNF-α or ER stress, which are reportedly increased in the joints of OA patients^4,5,10,11^. As TNF-α or ER stress has been shown to reduce intracellular ATP, we used MIA, a metabolic inhibitor, to mimic intracellular ATP depletion and subsequent cytotoxicity. When ATDC5-derived chondrocytes were treated with MIA, the intracellular ATP concentration rapidly decreased. Probably, the ability of MIA to inhibit glycolysis and to reduce ATP is too strong; thus, we did not find any conditions under which KUS121 could suppress the decrease in ATP. In contrast, KUS121 protects against cell death in MIA-treated ATDC5 cells (Supplementary Fig. 4). Although the model is usually used for pain research, MIA-induced arthritis shows pathological and pharmacological features similar to those of human OA^19,20^. Using this model, we showed that intraperitoneal administration of KUS121 ameliorated the damage caused by mechanical injury to the knee joint. The OARSI score of the Safranin-O-stained knee joint was lower in the KUS121-treated group than that in the vehicle-treated control group.

In orthopedics, intra-articular administration is often used to apply drugs to the knee joint. It is safer because it requires a lower dose than systemic administration. The number of ssDNA-positive apoptotic cells in rat knee joints upon intra-articular KUS121 administration was lower than that in those upon intraperitoneal KUS121 administration. Therefore, to treat OA, intra-articular administration of KUS121 may be preferred.

The limitations of this study are as follows: first, we used only ATDC5-derived chondrocytes in vitro. Other chondrocytes, including primary chondrocytes, should be used to examine the precise mechanism of chondrocyte protection by KUS121. Second, we used only the MIA-induced OA model to elucidate chondrocyte protection by KUS121. Third, we did not perform experiments to elucidate the dose and time dependency of KUS121 administered intraarticularly.

We can conclude that KUS121 exerts cytoprotective effects on chondrocytes that have been damaged either by inflammation and/or by cell stress rather than by mechanical stress. Further research is needed to explain how KUS121 protects cells in OA in more detail. Nevertheless, our results demonstrate KUS121 as a promising therapeutic agent for OA.

## Methods

### Cell culture

ATDC5 mouse chondrogenic cell line was maintained in DMEM/Ham’s F12 medium (Nacalai Tesque, Kyoto, Japan) supplemented with 5% fetal bovine serum (Thermo Fisher Scientific, Waltham, MA, USA), 100 U/ml kanamycin (FUJIFILM Wako Pure Chemical, Osaka, Japan) in a humidity incubator at 37 °C with 5% CO_2_. For the induction of chondrocyte differentiation, insulin (10 μg/ml), transferrin (10 μg/ml), and sodium selenite (3 × 10^–8^ M) (FUJIFILM Wako Pure Chemical) were added to the medium^8,9^.

Cells were plated on a 12-well plate at a density of 4 × 10^4^ cells/well and used 14–19 days after the induction of chondrocyte differentiation. The medium was changed every other day. Differentiated ATDC5 cells were cultured with KUS121 (0 [DMSO], 50, 100 μM) in the presence of TNF-α (R&D Systems, Minneapolis, MN, USA, 10, 20 ng/ml) or TM (Nacalai Tesque, 0.2 or 3 μg/ml) or under no glucose conditions.

### Cell viability assay

Cell viability was assessed using the water-soluble tetrazolium (WST) assay. Briefly, after being exposed to various stresses including TNF-α, TM and glucose starvation, cells were incubated with the cell counting reagent SF (Nacalai Tesque) at 37 °C for 20 min. The absorbance of the supernatant was measured at 450 nm using an ARVO multi-label plate reader (Perkin Elmer, Waltham, MA, USA).

### ATP assay

ATP levels in ATDC5 cells were detected via the intracellular ATP assay Kit (Toyo B-net, Tokyo, Japan) using the luciferase luminescence method according to the manufacturer's protocol. Briefly, the supernatant was collected after adding cell lysis buffer to each well of cultured chondrocytes. A Luciferase luminescence reagent was added to the supernatant and the luminescence signal was measured using a Nivo multi-label plate reader (PerkinElmer). The total protein content of each well was determined using the Protein Assay BCA Kit (Nacalai Tesque). The luminescence signal was standardized to the total protein content.

### Quantitative real-time RT-PCR

Total RNA was extracted from differentiated ATDC5 cells using an RNeasy mini kit (QIAGEN, Hilden, Germany). RNA samples were treated with DNase I and reverse-transcribed into cDNA using a SuperScript III kit (Invitrogen, Carlsbad, CA, USA) according to the manufacturer’s protocol. Real-time PCR was carried out using the TB Green Premix Ex Taq II (Takara, Kusatsu, Shiga, Japan) and Stratagene Mx3000P (Agilent Technologies, Santa Clara, CA, USA). The primers used are as follows; *Sox9* forward, 5′AACATGGAGGACGATTGGAG3′; *Sox9* reverse, 5′TCCCCTCAAAATGGTAATGAG3′; *Col2a1* forward, 5′AGAACAGCATCGCCTACCTG3′; *Col2a1* reverse, 5′CTTGCCCCACTTACCAGTGT3′; Glyceraldehyde-3-phosphate dehydrogenase (*GAPDH*) forward, 5′TGTGTCCGTCGTGGATCTGA3′; *GAPDH* reverse, 5′TTGCTGTTGAAGTCGCAGGAG3′. The housekeeping *GAPDH* was used as an internal standard.

### Western blotting

Cells were lysed with radioimmunoprecipitation lysis buffer containing protease inhibitors. Protein concentration was determined using a Protein Assay BCA Kit (Nacalai Tesque). Equal aliquots of protein samples were separated on 10–20% SuperSep Ace (FUJIFILM Wako Pure Chemical) and transferred onto polyvinylidene fluoride membranes. The blot was cut to sizes of 100, 75, 50, 37, and 25 kDa in Fig. 1F. The blot of ≥ 100 kDa was used for collagen staining, the blot of 75–100 kDa was for Grp78 staining, the blot of 50–75 kDa was for MMP-13, and the blot of 25–37 kDa was for CHOP staining, although these were not used in this study. In Fig. 2E, the blot was cut to sizes of 100, 75, 50, 37, and 25 kDa. The blot of ≥ 100 kDa was used for collagen staining, the blot of 75–100 kDa was for Grp78 staining, and the blot of 25–37 kDa was for CHOP staining, although these were not used in this study. The membranes were incubated with primary antibodies including anti-cCasp-3 (1:1000, Cell Signaling Technology, Danvers, MA, USA), anti-CHOP (1:1000, Proteintech, Rosemont, IL, USA), anti-Grp78 (1:1000, Cell Signaling Technology), and anti-β-actin (1:5000, Sigma, St Louis, MO, USA) at 4 °C overnight. The membranes were incubated with secondary antibodies at room temperature for 1 h. Protein bands were detected using ECL Prime Western Blotting Detection Reagent (Cytiva, Tokyo, Japan) and visualized using a Molecular Imager ChemiDoc XRS + system (Bio-Rad, Hercules, CA, USA).

### Flow cytometry analysis of Annexin V staining

Cells were harvested with trypsin/ethylenediaminetetraacetic acid and stained using the Annexin V-FITC Apoptosis Detection Kit (BioVision, Milpitas, CA, USA). According to the manufacturer’s instructions, 500 μL of 1× binding buffer and 5 μL Annexin V-FITC were added to the 1–5 × 10^5^ collected cells which were then treated at room temperature in the dark for 5 min. FITC-positive apoptotic cells were detected using FACS Calibur (Becton Dickinson, Franklin Lakes, NJ, USA).

### Animal experiments

All protocols were approved by the Institutional Review Board of Kyoto University Graduate School of Medicine (MedKyo 17274, 18302, 19300, 20265). All animal care and experiments were conducted following the institutional guidelines of our Animal Committee.

Male Sprague–Dawley rats (SLC, Hamamatsu, Shizuoka, Japan) at 8 weeks of age were used for the study. Rats were placed in a room with normal lighting (12:12 h light–dark cycle) and controlled temperature conditions (22 °C). MIA (Sigma) was dissolved in saline. Under anesthesia via intraperitoneal injection of ketamine and xylazine, the knee joint was fixed at a bent position. MIA (0.5 mg/25 µL) was intra-articularly injected into the knee joint through the patellar tendon^20^. KUS121 was dissolved in 5% Cremophor EL (Sigma)/phosphate-buffered saline to obtain a 5 mg/mL solution. The dosage of intraperitoneal injection of KUS121 was determined in accordance with our previous experiments^16^. Six rats were intraperitoneally administered with KUS121 (50 mg/kg/day)^13^ (MIA + KUS121) or vehicle (MIA + vehicle), respectively, every day from 4 days before until 14 days after MIA injection.

For intra-articular administration, 25 μL of KUS121 dissolved in 5% D-glucose solution (500 µg/day) was administered. Rats were divided into four groups: MIA + vehicle (N = 5), MIA + IP KUS121 (N = 5), MIA + IA KUS121 (N = 5), MIA + IP and IA KUS121 (N = 5).

### Histological examination

Knee joints were fixed in 10% formalin for 7 days, decalcified in Morse solution for 7 days and then embedded in paraffin wax. The specimens were sectioned sagittally at 4 μm and stained with Safranin-O (Sigma) and Fast green (Sigma). Histological sections were visualized using a BZ900 microscope (KEYENCE, Osaka, Japan). Histological scores (cartilage damage and Safranin-O loss) were assessed according to the OARSI scoring^27^ by three blinded observers (H. M., M. S., and Y. I.).

### Immunohistochemical staining

Endogenous peroxidase activity was blocked using 0.3% H_2_O_2_ in methyl alcohol for 30 min after deparaffinization and antigen retrieval. Thereafter, an anti-ssDNA antibody (1:600, IBL, Fujioka, Gunma, Japan) was applied overnight at 4 °C. Samples were incubated with biotinylated secondary antibody, diluted 1:300, for 40 min. Avidin–biotin–peroxidase complex (ABC-Elite, Vector Laboratories, Burlingame, CA, USA), diluted 1:100, was then applied for 50 min. After washing, the H_2_O_2_ activity was detected with 3,3′-diaminobenzidine tetrahydrochloride, and the nucleus was counterstained with hematoxylin^28^. The ratio of ssDNA-positive cells was calculated by dividing the number of ssDNA-positive cells by the total number of chondrocytes above the Tidemark.

### Statistical analysis

Data were presented as the mean ± standard deviation. Statistical analysis was performed using SPSS (version 22, IBM, Armonk, NY). A Dunnett T3 test was used to compare parameters with multiple conditions in ATDC5 cells. A Mann–Whitney U test was performed according to the OARSI score. The level of statistical significance was set at p < 0.05.

## Supplementary Information

Supplementary Information.

## Data Availability

All data generated or analyzed data during this study are included in this published article and the supplementary information. This study follows the recommendations in the ARRIVE guidelines.
