# Supplementary material for: KUS121 attenuates the progression of monosodium iodoacetate-induced osteoarthritis in rats
Source: Sci Rep. 2021 Aug 2;11:15651. doi: 10.1038/s41598-021-95173-6 (PMC8329178; doi:10.1038/s41598-021-95173-6)
Supplement: Supplementary file 1 — Supplementary Information. [file 41598_2021_95173_MOESM1_ESM.pdf]

Supplementary Information

KUS121 attenuates the progression of monosodium iodoacetate-induced osteoarthritis in rats

Sachiko Iwai, Hanako O. Ikeda, Hisashi Mera, Kohei Nishitani, Motoo Saito, Akitaka Tsujikawa and Akira Kakizuka

A

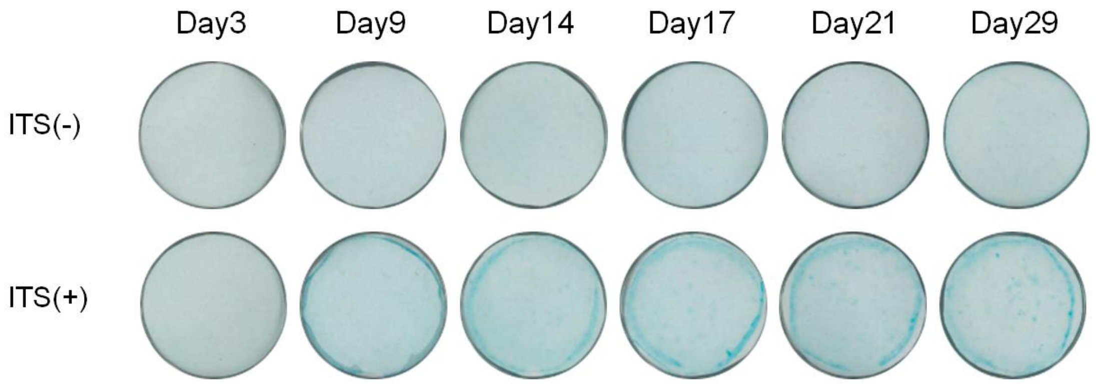

B

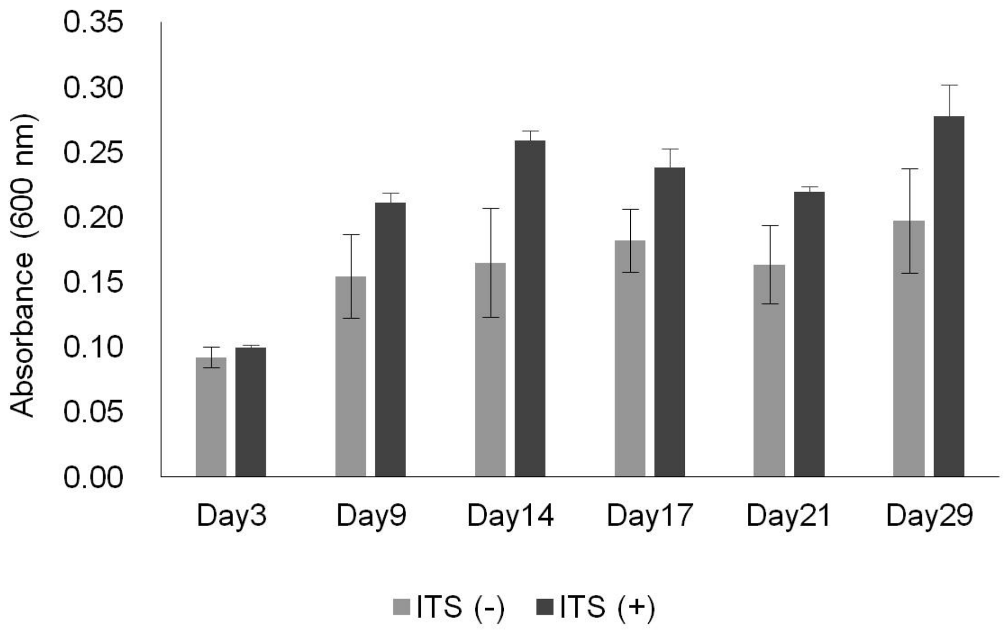

Supplementary Figure 1 Confirmation of cartilage differentiation in differentiated ATDC5 cells.

(A) Cells were stained with Alcian blue to detect cartilage matrix containing mucopolysaccharides 3, 9, 14, 17, 21, and 29 days after the start of differentiation using insulin, transferrin, and sodium selenite (ITS+ or ITS-). **(B)** The dye was removed with guanidine hydrochloride and the absorbance was measured at 600 nm. N = 3. Error bars indicate standard deviation.

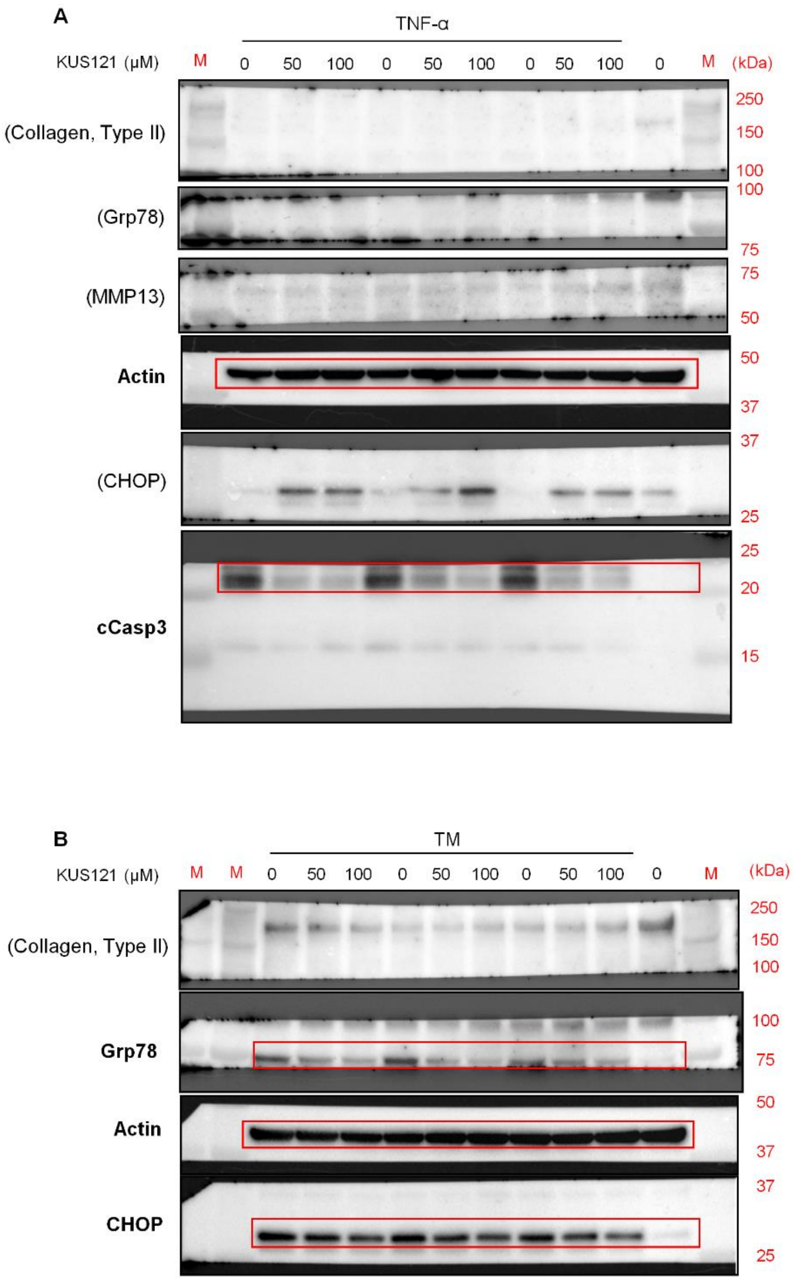

Supplementary Figure 2 Complete scans of the gels presented in Figure 1F and 2E

(A) The blot was cut to sizes of 100, 75, 50, 37, and 25 kDa. The blot of ≥ 100 kDa was used for collagen staining, the blot of 75–100 kDa was for Grp78 staining, the blot of 50–75 kDa was for MMP-13, and the blot of 25–37 kDa was for CHOP staining, although these were not used in this study. Actin and cCaspase staining results are shown in Fig. 1F. **(B)** The blot was cut to sizes of 100, 75, 50, 37, and 25 kDa. The blot of ≥ 100 kDa was used for collagen staining, the blot of 75–100 kDa was for Grp78 staining, and the blot of 25–37 kDa was for CHOP staining, although these were not used in this study. M, protein molecular weight marker; TNF-α, tumor necrosis factor-alpha; TM, tunicamycin.

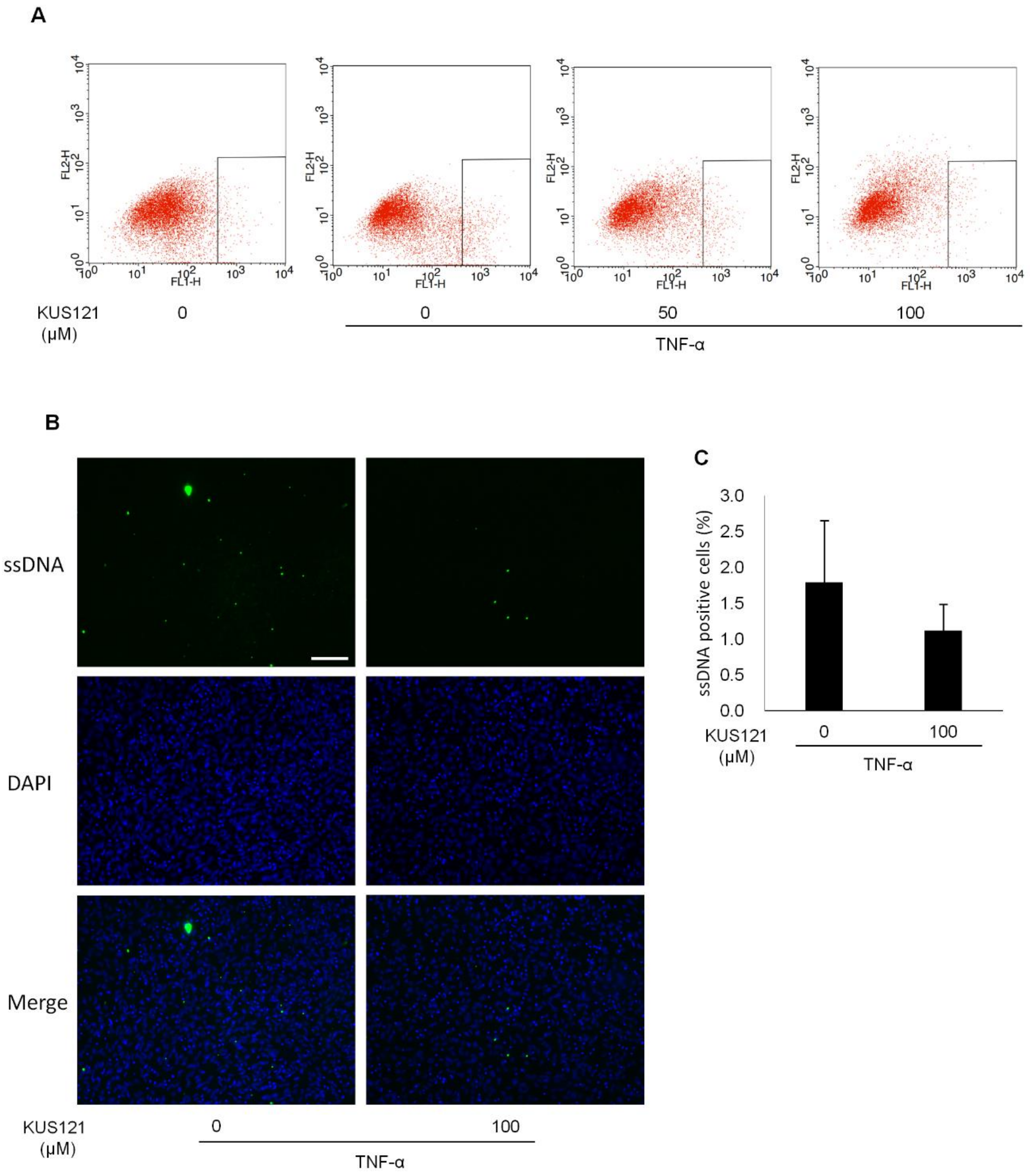

Supplementary Figure 3 Staining for detection of apoptotic cells in differentiated ATDC5 cells

(A-C) Differentiated ATDC5 cells cultured with tumor necrosis factor-alpha (TNF-α, 20 ng/mL) and KUS121 (0, 50 or 100 μM) for 48 h. **(A)** Cells were stained with a FITC-conjugated annexinV antibody and detected via fluorescence activated cell sorting. The number of cells in the boxed area were measured. **(B, C)** Cells were stained with an anti-single strand DNA (ssDNA) antibody (green) and 4',6-diamidino-2-phenylindole (DAPI, blue). Scale bar, 100 μm. **(C)**. The ratio of the number of ssDNA-positive cells to the total number of DAPI-stained cells are shown. Error bars indicate standard deviation.

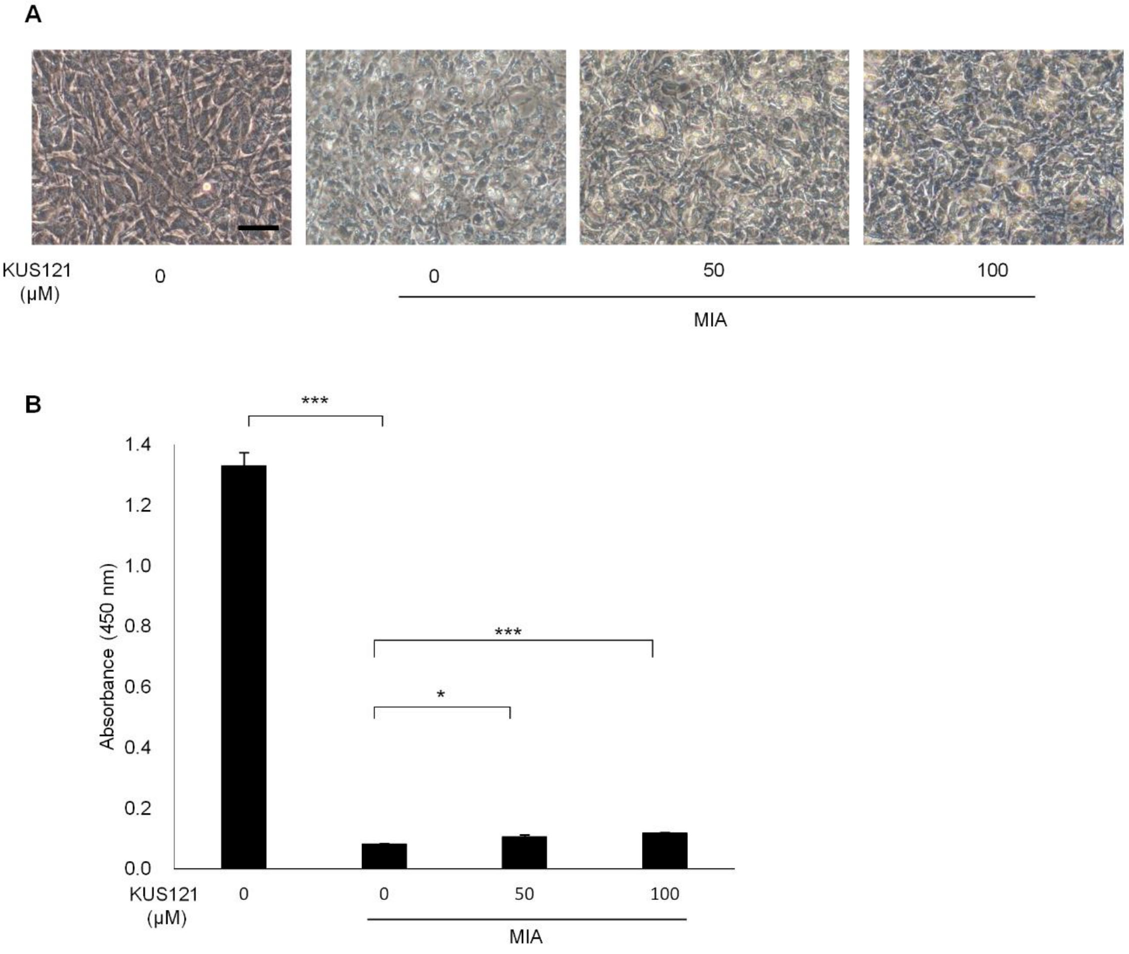

Supplementary Figure 4 KUS121 suppresses monosodium iodoacetate-induced cell death in differentiated ATDC5 cells

(A, B) Differentiated ATDC5 cells cultured with monosodium iodoacetate (MIA, 0.1 mg/mL) and KUS121 (0, 50 or 100 µM) for 24 h. Brightfield images of the cells. Scale bar, 50 µm. **(B)** Water-soluble tetrazolium salt values, which reflect the relative live cell number, are shown as optical density at 450 nm. *** $p < 0.001$, * $p < 0.05$, vs MIA without KUS121, Dunnett T3 test, N = 4. Error bars indicate standard deviation.

Supplementary methods

Histochemistry

ATDC5 cells were seeded onto 12-well plates and cultured for the indicated periods (Days 3, 9, 14, 17, 21, and 29). For Alcian blue staining, cells were fixed with 95% methanol for 20 min and stained with 0.1% Alcian blue 8GS (Sigma) in 0.1 M HCl overnight and rinsed with distilled water. The Alcian blue dye was removed with 6 M guanidine hydrochloride (Sigma) for 6 h. The absorbance of the extracted dye was measured at 600 nm.

Immunofluorescence staining

Cells were fixed with 4% paraformaldehyde and permeabilized with Triton-X. The sample was then incubated with 10% goat serum to block specific sites and incubated overnight at 4 °C with an ssDNA antibody (1: 50, IBL). After washing, cells were incubated with an Alexa Fluor 488-conjugated secondary antibody (Thermo Fisher Scientific) and DAPI at 37 °C for 1 h. Images were taken using a BZ900 microscope (KEYENCE).
